# A European Pathogenic Microorganism Proteome Database: Construction and Maintenance

**DOI:** 10.1002/cfg.165

**Published:** 2002-04

**Authors:** Klaus-Peter Pleißner, Till Eifert, Peter R. Jungblut

**Affiliations:** 1 Max Planck Institute for Infection Biology Core Facility Bioinformatics Berlin Germany; 2 Algorithmus Berlin Germany; 3 Max Planck Institute for Infection Biology Core Facility Protein Analysis Schumannstr. 20–21 Berlin D-10117 Germany

## Abstract

A relational database structure based on MS-Access and MySQL to store and manage proteomics data was established. This system may be used to publish two-dimensional electrophoretic proteomics data, and also may be accessed by external users who want to compare their own data with those in the databases. The maintenance of the database is managed centrally. The producers of proteomics data do not need to construct a database themselves. Users can introduce mass spectra into the database, which allows the searching of peptide mass fingerprints against their own protein sequence databases. The first release published in January 2002 contains data from *Mycobacterium tuberculosis, Helicobacter pylori, Borrelia garinii, Francisella tularensis, Chlamydia pneumoniae, Mycoplasma pneumoniae*, Jurkat T-cells and mouse mammary gland projects (http://www.mpiib-berlin. mpg.de/2D-PAGE/).


**Comparative and Functional Genomics**
*Comp Funct Genom* 2002; **3**: 97–100.
Published online 12 March 2002 in Wiley InterScience (www.interscience.wiley.com). **DOI:** 10.1002/cfg.165

**Short Communication**

### A European pathogenic microorganism proteome database: construction and maintenance

Klaus-Peter Pleißner[1], Till Eifert[2] and Peter R. Jungblut[3]*

[1] *Max Planck Institute for Infection Biology, Core Facility Bioinformatics, Berlin, Germany*
[2] *Algorithmus, Berlin, Germany*
[3] *Max Planck Institute for Infection Biology, Core Facility Protein Analysis, Berlin, Germany*

*Correspondence to: Max Planck Institute for Infection Biology, Core Facility Protein Analysis, Schumannstr. 20–21, D-10117, Berlin, Germany. E-mail: jungblut@mpiib-berlin.mpg.de*

Received: 18 February 2002
Accepted: 19 February 2002

### Abstract

**A relational database structure based on MS-Access and MySQL to store and manage proteomics data was established. This system may be used to publish two-dimensional electrophoretic proteomics data, and also may be accessed by external users who want to compare their own data with those in the databases. The maintenance of the database is managed centrally. The producers of proteomics data do not need to construct a database themselves. Users can introduce mass spectra into the database, which allows the searching of peptide mass fingerprints against their own protein sequence databases. The first release published in January 2002 contains data from *Mycobacterium tuberculosis*, *Helicobacter pylori*, *Borrelia garinii*, *Francisella tularensis*, *Chlamydia pneumoniae*, *Mycoplasma pneumoniae*, Jurkat T-cells and mouse mammary gland projects (http://www.mpiib-berlin.mpg.de/2D-PAGE/). Copyright © 2002 John Wiley & Sons, Ltd.**

**Keywords:** **proteomics; database; WWW; microorganism; mass spectrometry; two-dimensional electrophoresis**

### Introduction

The vast amount of data generated by proteomics studies and the need for its effective storage, interpretation and exchange makes informatics methods for data basing and data mining essential. Since the beginning of the nineties the number of databases for molecular biological data has increased exponentially. A comprehensive overview of such databases concerning genomics, transcriptomics and proteomics data is published in the January issue of Nucleic Acids Research each year. For classical proteomics, combining two-dimensional gel electrophoresis (2-DE) with identification methods such as mass spectrometry or Edman sequencing, specialized 2-DE databases have also been constructed. Many of these databases are located within the WORLD-2DPAGE database (**http://www.expasy.ch/ch2d/2d-index.html**). Besides the well known public sequence databases available from NCBI or other bioinformatics facilities, the WWW accessible 2-DE databases form the link between descriptive (textual) information of proteins and the image information (2-DE gels) showing the entire protein patterns of tissues, cells, cell compartments, body fluids or microorganisms. These proteome patterns can be interpreted as a snapshot of proteins in different organisms, states or time courses. The comparison of different proteomes, for instance by computerized gel image analysis, further facilitates the assessment of changes at the protein species level. The results of such comparisons need to be presented in 2-DE databases to depict the dynamic changes in proteome patterns under different conditions. Currently, about forty 2-DE databases exist and the most important are indexed at the ExPASy molecular biology server. The overall principles and rules of construction of such databases were described elsewhere (Appel *et al.*, 1993; Appel *et al.*, 1996). The freely available software tool make2ddb (**http://www.expasy.org/ch2d/make2ddb.html**) allows the construction of federated 2-DE databases standardized by several rules.

Here we present a relational database and all of

Copyright © 2002 John Wiley & Sons, Ltd.

the tools necessary to construct a proteome database, including free downloadable programs to prepare gel images for the database. The standardized database is managed centrally, keeping the data even after the investigators have changed their research interests. A main focus of this database will be pathogenic microorganisms, primarily organized within the European pathogenic microorganism database. This database fulfils the guidelines for building a federated 2-DE database and therefore is listed in the WORLD-2DPAGE index.

### Material and methods

### 2D-PAGE database

The 2D-PAGE proteome database (**http://www.mpiib-berlin.mpg.de/2D-PAGE/**) began as a mycobacterial 2-DE database (Mollenkopf *et al.*, 1999). As an alternative to the make2ddb tool this database is based on our own software developments and an extensive use of a relational database model. The program package consists of common gateway interface (CGI) scripts written in PERL and PHP, the GD graphic library (**http://www.boutell.com/gd/**) for manipulating images and the relational database management system MySQL (**http://www.mysql.com/**). Internet access is accomplished via an Apache Web server on a UNIX platform. The MS-Access database is used as a front-end for the acquisition of textual descriptive data. To facilitate the input of descriptive information, self-explanatory MS-Access templates were created. The relational structure of tables is defined using MS-Access and coincides with the database structure in MySQL.

The TopSpot gel image processing system is used to prepare the 2-DE gel images for database construction. This is a personal computer version of a program presented earlier (Prehm *et al.*, 1987). After scanning and fragmentation of gels into subsections, spot detection is performed yielding gif-images of each subsection, and corresponding MAP-files describing the positions by polygons of spot contours. The polygon approximations of protein spots serve as sensitive clickable areas within WWW-accessible gel images and provide links to the descriptive information. As depicted in Figure 1 the preparation of gel images and acquisition of descriptive data is carried out at the user level. Transferring the corresponding files (GIF-images, MAP-files and MS-Access database files) to the database administrator by email or file transfer protocol, a database is established without further user interactions at the administration level (Figure 1).

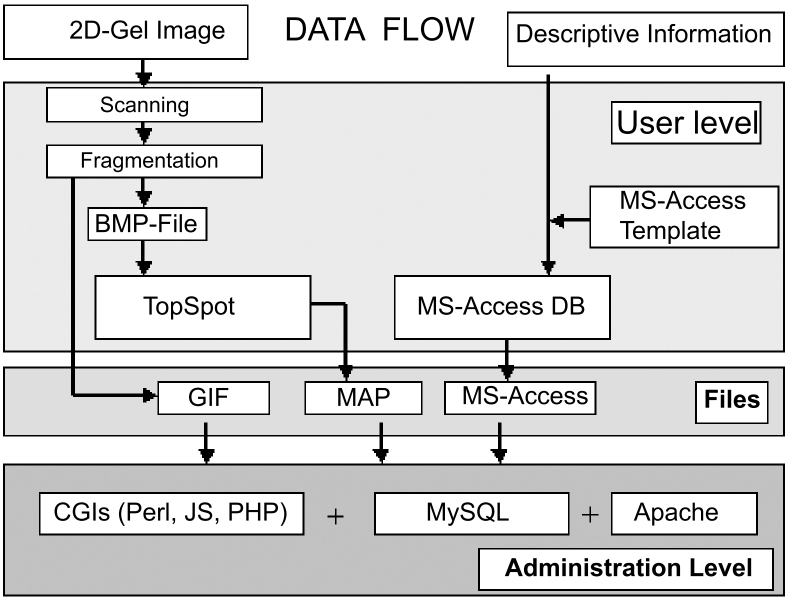

**Figure 1.** Flow chart of information processing during the construction of the pathogenic microorganism proteome 2-DE database

Copyright © 2002 John Wiley & Sons, Ltd. 

The entire software package including TopSpot, fragmentation, and MS-Access templates is running on the Windows platforms and is downloadable free of charge from the download area of the 2D-PAGE database website. In the downloaded version, all database structures are revealed; i.e. the field names, data types, table names and others are given. The user may define additional fields. The TopSpot program is easy to use and a README file is included explaining the application. An intranet version may also be created, which allows the database creating institution an exclusive access to the database until the data are released to the scientific community.

### Results and discussion

Since the first release of the mycobacterial 2D-PAGE database (Mollenkopf *et al.*, 1999) a multi-species database has evolved containing proteomic information on *Mycobacterium bovis*, *M. tuberculosis* (Jungblut *et al.*, 1999a, *Helicobacter pylori* (Jungblut *et al.*, 2000), *Borrelia garinii* (Jungblut *et al.*, 1999b), *Francisella tularensis* (Hernychova *et al.*, 2001), *Mycoplasma pneumoniae* (Ueberle *et al.*, 2001), *Chlamydia pneumoniae* (Vandahl *et al.*, 2001), mouse mammary gland (Aksu *et al.*, 2001) and human Jurkat T-cells (Thiede *et al.*, 2000). Additionally, several structural improvements were added to enhance the quality of data and data integrity.

Mass spectrometry data (peptide mass fingerprints) can now be included to comprehend the process of protein identification. Synthetic MS spectra are generated from the experimentally determined mass list and the five most intense peaks are marked. In addition the original mass spectra are downloadable to represent them in an original manner using the m/z program (**http://canada.proteometrics.com/Download/mz.html**) for a repeated analysis of protein mass spectra. Peaks not assigned in earlier investigations may now be re-interpreted and checked for post-translational modifications or point mutations.

The proteome database is the first step to collect and organize the data obtained by the classical proteome approach combining 2-DE and mass spectrometry. In a second step, functional information will be obtained from the database: Protein subsets and their positions on the 2-DE maps can be visualized. As an example the antigens of *Helicobacter pylori* can be shown on the gel image. Due to the relational organization of the database, multiple term/ multiple keyword searches such as '*show all antigens with a Mr between 20 and 30 kDa and with a pI value between 5 and 6*' will be possible in the near future. If protein subsets with different properties (antigens, Mr, pI, different locations, and different reactions to environmental changes) cluster, interrelationships between these subsets exist and will correlate functions with molecular changes at the protein level.

Some further formal improvements were introduced: the original 2-DE pattern may be subdivided into a variable number of image subsections. If large gels with a size of 30 cm × 40 cm are used, it is recommended to subdivide the gels into 12 sectors, whereas for 20 cm × 20 cm gels 4 sectors of 10 cm × 10 cm are sufficient for efficient gel evaluation and presentation. Molecular mass and iso-electric point calibrations are presented at the axes of the gel images. Within the subsections zooming-in is now available.

In contrast to flat file based databases, the relational approach enables a normalization of data to improve the data integrity and to decrease the redundancy. Moreover, a release mechanism was introduced to update the corresponding entries automatically. Currently, the 2D-PAGE proteome database at MPI for Infection Biology is serving as one of the working standards, together with the SWISS-2DPAGE standard, within the European network project on human pathogenic bacteria, EBP, (QLRT-1999-31536).

Using the software package presented above, three additional 2-DE databases were recently established for *F. tularensis* in cooperation with J. Stulik from the Czech Republic, for *M. pneumoniae* in cooperation with R. Herrmann in Heidelberg, Germany, and, within the EBP network project, with G. Christiansen in Aarhus, Denmark for *C. pneumoniae* as a supplement to their local PCHI-2DPAGE database at (**http://www.gram.au.dk/**). Descriptive information on proteins was given in EXCEL-sheets by the Danish group and imported into MS-Access templates by our database manager. The preparation of the data for database construction needs only some hours, if the guidelines within the README-file are followed strictly.

In order to combine protein functions with 2-DE data first attempts were undertaken to incorporate 2-DE *H. pylori* data into the protein extraction, description and analysis tool PEDANT (**http://**

Copyright © 2002 John Wiley & Sons, Ltd. 

**pedant.gsf.de/**) (Frishman *et al.*, 2001). For the integration of our database we intend to use the BioRS retrieval system allowing the integration of heterogeneous databases with in-house proprietary databases.

In conclusion, this freely downloadable software package including the TopSpot-program and the MS Access templates enables biologists to construct 2-DE databases without skilled bioinformatic knowledge, because the administrator provides database administration and maintenance tasks. Furthermore, the complete soft- and hardware comprising MS-Access and Windows PCs should be available in almost all molecular biological laboratories. We encourage interested users to apply our software package and give us feedback for further improvements.

### Acknowledgement

The construction of a European Bacterial Proteome Database is supported by European Union (EBP: QLRT-1999-31536) and the combination with PEDANT by BMBF (O31U107A and O31U207A). The authors wish to acknowledge SHE Kaufmann and TF Meyer for their continuous support to establish the database at the Max Planck Institute for Infection Biology.

### References

Aksu S, Scheler C, Focks N, *et al.* 2002. An iterative calibration method with prediction of posttranslational modifications for the construction of a two-dimensional electrophoresis database of mouse mammary gland proteins. *Proteomics* **2**: (submitted).

Appel RD, Sanchez JC, Bairoch A, *et al.* 1993. SWISS-2DPAGE: a database of two-dimensional gel electrophoresis images. *Electrophoresis* **14**: 1232–1238.

Appel RD, Bairoch A, Sanchez JC, *et al.* 1996. Federated two-dimensional electrophoresis database: a simple means of publishing two-dimensional electrophoresis data. *Electrophoresis* **17**: 540–546.

Frishman D, Albermann K, Hani J, *et al.* 2001. Functional and structural genomics using PEDANT. *Bioinformatics* **17**: 44–57.

Hernychova L, Stulik J, Halada P, *et al.* 2001. Construction of *Francisella tularensis* two-dimensional electrophoresis protein database. *Proteomics* **1**: 508–515.

Jungblut PR, Schaible UE, Mollenkopf H-J, *et al.* 1999a. Comparative proteome analysis of *Mycobacterium tuberculosis* and *Mycobacterium bovis* BCG strains: Towards functional genomics of microbial pathogens. *Mol Microbiol* **33**: 1103–1117.

Jungblut PR, Grabher G, Stöffler G. 1999b. Comprehensive detection of immunorelevant *Borrelia burgdorferi* antigens by two-dimensional electrophoresis. *Electrophoresis* **20**: 3611–3622.

Jungblut PR, Bumann D, Haas G, *et al.* 2000. Comparative proteome analysis of *H. pylori*. *Mol Microbiol* **36**: 710–725.

Mollenkopf HJ, Jungblut PR, Raupach B, *et al.* 1999. A dynamic two-dimensional polyacrylamide gel electrophorsis database: The mycobacterial proteome via Internet. *Electrophoresis* **20**: 2172–2180.

Prehm J, Jungblut PR, Klose J. 1987. Analysis of two-dimensional protein patterns using a video camera and a computer. *Electrophoresis* **8**: 562–572.

Thiede B, Siejak F, Dimmler C, *et al.* 2000. A two-dimensional electrophoresis database of a human Jurkat T cell line. *Electrophoresis* **21**: 2713–2720.

Ueberle B, Boguth G, Goerg A, *et al.* 2001. Proteome analysis of the bacterium *Mycoplasma pneumoniae* – an update. *Proteomics* **2**: (in press).

Vandahl BB, Birkelund S, Demol H, *et al.* 2001. Proteome analysis of the *Chlamydia pneumoniae* elementary body. *Electrophoresis* **22**: 1204–1223.

Copyright © 2002 John Wiley & Sons, Ltd.
